# The role of RhoC in epithelial-to-mesenchymal transition of ovarian carcinoma cells

**DOI:** 10.1186/1471-2407-14-477

**Published:** 2014-07-01

**Authors:** Wen-feng Gou, Yang Zhao, Hang Lu, Xue-feng Yang, Yin-ling Xiu, Shuang Zhao, Jian-min Liu, Zhi-tu Zhu, Hong-zhi Sun, Yun-peng Liu, Feng Xu, Yasuo Takano, Hua-chuan Zheng

**Affiliations:** 1 Cancer Research Center, The First Affiliated Hospital of Liaoning Medical University, 121001 Jinzhou, China; 2 Key Laboratory of Brain and Spinal Cord Injury of Liaoning Province, The First Affiliated Hospital of Liaoning Medical University, 121001 Jinzhou, China; 3 Department of Gynecology, The First Affiliated Hospital of China Medical University, 110001 Shenyang, China; 4 Department of Oncological Medicine, The First Affiliated Hospital of China Medical University, 110001 Shenyang, China; 5 Department of Physiology, School of Life Science and Biopharmaceutics, Shenyang Pharmaceutical University, 110016 Shenyang, China; 6 Clinical Research Institute, Kanagawa Cancer Center, 241-0815 Yokohama, Japan

**Keywords:** Ovarian carcinoma, RhoC, Epithelial-to-mesenchymal transition

## Abstract

**Background:**

RhoC is a small G protein/GTPase and involved in tumor mobility, invasion and metastasis. Previously, up-regulated RhoC expression is found to play an important role in ovarian carcinogenesis and subsequent progression by modulating proliferation, apoptosis, migration and invasion.

**Methods:**

We transfected RhoC-expressing plasmid and RhoC siRNA into CAOV3 and OVCAR3 cells respectively. These cells and transfectants were exposed to vascular epithelial growth factor (VEGF), transforming growth factor (TGF)-β1 or their receptor inhibitors with the phenotypes and their related-molecules examined.

**Results:**

TGF-β1R or VEGFR inhibitor suppressed the proliferation, migration, invasion and lamellipodia formation, the expression of N-cadherin, α-SMA, snail and Notch1 mRNA or protein, and enhanced E-cadherin mRNA and protein expression in CAOV3 and its RhoC-overexpressing transfectants, whereas both growth factors had the opposite effects in OVCAR3 cells and their RhoC-hypoexpressing transfectants. Ectopic RhoC expression enhanced migration, invasion, lamellipodia formation and the alteration in epithelial to mesenchymal transition (EMT) markers of CAOV3 cells regardless of the treatment of VEGFR or TGF-β1R inhibitor, whereas RhoC knockdown resulted in the converse in OVCAR3 cells even with the exposure to VEGF or TGF-β1.

**Conclusion:**

RhoC expression might be involved in EMT of ovarian epithelial carcinoma cells, stimulated by TGF-β1 and VEGF.

## Background

Ovarian cancer is the second leading cancer in women and the 5th leading cause of cancer-related deaths in women [1]. Ovarian cancer is disproportionately deadly because no sophisticated approach for the early diagnosis makes most ovarian cancers diagnosed at advanced stages, which determines the five-year survival rate of ovarian cancer comparatively low [2]. The existence of cancer stem-like cells from epithelial to mesenchymal transition (EMT) makes ovarian cancer more frequently recurrent and drug-resistant [3].

EMT is a process that epithelial cells are converted from a phenotypic shift from cells with tight cell–cell junctions, clear basal and apical polarity, and sheet-like growth architecture into spindle-like and motile cells, which is associated with cancer progression, cell invasion, chemotherapeutic resistance and the formation of side populations of cancer stem-like cells [4]. EMT is triggered by the interplay of extracellular signals (collagen, hyaluronic acid and integrin), such secreted factors as transforming growth factor (TGF)-β, vascular endothelial growth factor (VEGF), epithelial growth factor, hepatocyte growth factor, Wnt proteins and matrix metalloproteinases. The receptor-mediated signal pathways involve Akt, glycogen synthase kinase-3, Rho-GTPases and Smad, finally to up-regulate a set of transcription factors including Snai1, Slug, Zeb1, Zeb2, Goosecoid, and forkhead box protein C2, which regulate the expression of epithelial and mesenchymal markers at a transcriptional level [4-6]. Consequently, there appear down-regulation of epithelial markers (E-cadherin, desmoplakin and plakoglobin) and up-regulation of mesenchymal markers (N-cadherin, fibronectin and α-SMA). E-cadherin loss might lead to the disruption of cell-cell adhesion and the translocation of β-catenin into the nucleus [4].

Reportedly, either up-regulation or increased activity of RhoC promotes the invasive potential of cancer cells, which is closely associated with EMT [7]. RhoC is a small (~21–25 kDa) G protein/GTPase which belongs to the Rac subfamily of Rho family. It shuttles between inactive GDP-bound and active GTP-bound states and serves as a molecular switch in signal transduction cascades [8]. It has been found that RhoC promotes reorganization of the actin cytoskeleton, regulates cell shape and attachment, and coordinates cell motility and actomyosin contractility. RhoC overexpression is associated with cell invasion and metastasis of ovarian cancer [9,10]. RhoC-deficient mice can still develop tumors, which however fail to metastasize, arguing that RhoC is essential for metastasis [11]. In cervical carcinoma cells, both Notch1 and RhoC have similar phenotypic contribution to EMT, and Notch1 inhibition decreases RhoC activity, suggesting that RhoC functions as an effector of Notch1 [12]. Sequeira et al. [13] demonstrated that RhoC inactivation resulted in morphological changes of mesenchymal to epithelial transition and was accompanied by decreased direct migration and invasion of human prostate cancer cells. Bellovin et al. [14] reported that RhoC expression and activation are induced by EMT of colon carcinoma cell and RhoC promotes post-EMT cell migration.

Previously, we found that the RhoC mRNA and protein were significantly higher in ovarian cancer, and correlated with clinicopathological staging [9]. The RhoC knockdown resulted in a low growth, G_1_ arrest, apoptotic induction of OVCAR3 cells with the decreased expression of *Akt*, *stat-3*, *bcl-xL* and *survivin,* and the increased expression of *Bax* and *Caspase-3*[10]. Here, we aimed to clarify the role of RhoC in EMT process of ovarian carcinoma, stimulated by TGF-β1 and VEGF.

## Methods

### Plasmid construction

RhoC was amplified using the template of OVCAR3 cDNA and inserted into pBluescript-K by *Hinc* II. The primers of RhoC were forward: 5′- CCGGAATTCATGGCTGCAATCCGA AA-3′ and reverse: 5′-CGCGGATCCTCAGAGAATGGGACAGC-3′. Target RhoC DNA was digested and inserted into pEGFP-N1 between *EcoR* I and *BamH* I.

### Cell culture and transfection

Ovarian carcinoma cell lines, CAOV3 (serous adenocarcioma), OVCAR3 (serous cystic adenocarcinoma), SKOV3 (serous papillary cystic adenocarcinoma), HO8910 (serous cystic adenocarcinoma), and ES-2 (clear cell carcinoma) have been purchased from ATCC. They were maintained in RPMI 1640 (ES-2, HO8910 and OVCAR3), DMEM (CAOV3) and McCoy's 5A (SKOV3) medium supplemented with 10% fetal bovine serum (FBS), 100 units/mL penicillin, and 100 μg/mL streptomycin in a humidified atmosphere of 5% CO_2_ at 37°C.

The ovarian carcinoma cells were treated with *RhoC*-expressing plasmid by Attractene Transfection Reagent (QIAGEN) with pEGFP-N1 as a mock or RhoC siRNA (Sigma, USA) by HiPerFect Transfection Reagent (QIAGEN). The target sequences of RhoC siRNA were 5′-GUGCCUUUGGCUACCUUGAdTdT-3′ (sense) and 5′-UCAAGGUAGCCAAAGGCA CdTdT-3′ (anti-sense). The negative siRNA control sequences were 5′-UUCUCCGAACGU GUCACGUT T-3′ (sense) and 5′-ACGUGACACGUUCGGAGAATT-3′ (anti-sense). Cells were treated by recombinant human TGF-β1 and VEGF165 (Perotech), VEGF receptor inhibitor BIBF1120 and TGF-β1 receptor inhibitor SB431542 (Selleckchem). All cells were harvested by centrifugation, rinsed with phosphate buffered saline (PBS), and subjected to RNA and protein extraction.

### Proliferation assay

Cell counting Kit-8 (CCK-8, Japan) was employed to determine the number of viable cells. In brief, 2.5 × 10^3^ cells/well were seeded on 96-well plate and allowed to adhere. At different time points, 10 μL of CCK-8 solution was added into each well of the plate and the plates were incubated for 3 h and measured at 450 nm.

### Wound healing assay

Cells were seeded at a density of 1.0 × 10^6^ cells/well in 6-well culture plates. After they had grown at the confluence of 70-80%, the cell monolayer in each well was scraped with a pipette tip to create a scratch, washed by PBS for three times and cultured in the FBS-free medium. Cells were photographed at 48 h and the scratch area was measured using Image software.

### Cell invasion assays

For invasive assay, 2.5 × 10^5^ cells were resuspended in serum-free DMEM or RPMI 1640 medium, and seeded in the matrigel-coated insert on the top portion of the chamber (Corning). The lower compartment of the chamber contained 10% FBS as a chemoattractant. After incubated at 37°C and 5% CO_2_ for 24 h, filter inserts were removed from the wells. Cells on the upper surface of the filter were removed using a cotton swab. Those on the lower surface were fixed with 20% methanol in PBS, stained with Giemsa dye for the measurement.

### Immunofluorescence

Cells were grown on glass coverslips and treated as described in the figure legends. Cells were washed twice with PBS, fixed with 4% formaldehyde for 10 min, and permeabilized with 0.2% Triton X-100 for 10 min. After washing with PBS, cells were incubated overnight at 4°C with the rabbit antibody against E-cadherin (Abcam) and the mouse antibody against N-cadherin (Abcam). They were then washed with PBS, and incubated with anti-mouse Alexa Fluor 594 (red) IgG and anti-rabbit Alexa Fluor 488 (green) IgG (Invitrogen). Alexa Fluor® 594 phalloidin (red, invitrogen) for F-actin staining was employed to observe the lamellipodia. Nuclei were stained with 1 μg/mL DAPI (Sigma) for 30 min at 37°C. Finally, coverslips were mounted with SlowFade® Gold antifade reagent (invitrogen) and observed under laser confocal scanning microscope (Leica). Densitometric quantification of protein immunoreactivity was performed using Image-pro plus software (Media Cybernetics, Netherlands).

### Real-time RT-PCR

Total RNA was extracted from ovarian carcinoma cell lines using Trizol (Takara, Japan) according to the manufacturer’s protocol. Two micrograms of total RNA was subjected to cDNA synthesis using AMV reverse transcriptase and random primer (Takara, Japan). According to Genbank, oligonucleotide primers for PCR were designed and shown in Table 1. Real-time PCR amplification of cDNA was performed in 20 μL mixtures according to the protocol of SYBR Premix Dimer Eraser kit (Takara) with GAPDH as an internal control. The expression level was expressed as 2^-∆Ct^, where ∆Ct = Ct (gene) - Ct (GAPDH). Additionally, the expression level of the control cells was considered as “1”.

**Table 1:** Primers’ design for RT-PCR

**Names**	**Primer‘s sequence**	**Distribution**	**AT (°C)**	**Product size(bp)**	**Extension time(s)**
*N-cadherin*	F:5′-GAAAGACCCATCCACG- 3′	NM-031333.1	60	217	34
	R: 5′-CCTGCTCACCACCACTA- 3′	2365-2581			
*E-cadherin*	F:5′-CCGCCATCGCTTACA-3′	NM-057374.2	60	262	34
	R:5′-GGCACCTGACCCTTGTA-3′	1017-1278			
*a-SMA*	F:5′-GAGCGTGAGATTGTCCG-3′	NM-007392.2	60	232	34
	R: 5′-TGCTGTTGTAGGTGGTTTC-3′	583-814			
*Snail*	F:5′-GGCTCAGTTCGTAAAGG-3′	NM-001032543.1	60	357	34
	R:5′-GCAGCGGTAGTCCACA-3′	7-363			
*Slug*	F:5′-ATGCCTGTCATACCACAA-3′	FBgn0028564	60	173	34
	R: 5′-GAGGAGGTGTCAGATGGA-3′	290-462			
*RhoC*	F:5′-TGCCTCCTCATCGTCTTCA-3′	NM-001042678.1	60	310	34
	R: 5′-GCCTCAGGTCCTTCTTATTCC-3′	391-700			
*GAPDH*	F: 5′-CAATGACCCCTTCATTGACC-3′	NM_ 002046.3	60	135	34
	R: 5′- TGGAAGATGGTGATGGGATT-3′	201-335			

AT = annealing temperature.

### Western blot

Total protein was extracted by sonication in radioimmunoprecipitation assay(RIPA) buffer (50 mM Tris–HCl pH 7.5, 150 mM NaCl, 5 mM EDTA, 0.5% Nonidet P-40, 5 mM dithiothreitol, 10 mM NaF, protease inhibitor cocktail). One hundred or seventy μg denatured protein was separated on an SDS-polyacrylamide gel and transferred to Hybond membrane (Amersham, Germany), which was then blocked overnight in 5% skim milk in tris buffered saline with Tween 20 (TTBS, 10 mM Tris–HCl, 150 mM NaCl, 0.1% Tween 20). For immunobloting, the membrane was incubated for 15 min with the primary antibody (Table 2). Then, it was rinsed by TBST and incubated with anti-mouse, anti-rabbit or anti-goat IgG conjugated to horseradish peroxidase (DAKO, USA, 1:1000) for 15 min. All the incubations were performed in a microwave oven to allow intermittent irradiation [15]. Bands were visualized with LAS4010 (GE healthcare Life Science, USA) by ECL-Plus detection reagents (Santa Cruz, USA). After that, membrane was washed with WB Stripping Solution (pH2-3, Nacalai, Tokyo, Japan) for 1 h and treated as described above except mouse anti-GAPDH antibody (Sigma, 1:10,000). Densitometric quantification of protein bands was performed with GAPDH as an internal control using Image J (NIH, USA).

**Table 2:** Antibodies’ used in Western blot

**Names**	**Species**	**MW**	**Dilution**	**Code**	**Source**
E-Cadherin	Rabbit	97 kDa	1:1000	ab53033	abcam, USA
N-Cadherin	mouse	100 kDa	1:1000	ab98952	abcam, USA
α-SMA	mouse	42 kDa	1:1000	ab3280	abcam, USA
Slug	rabbit	30 kDa	1:1000	ab27568	abcam, USA
Notch1	goat	300KD	1:500	sc-6014	Santa cruz, USA
RhoC	goat	24KD	1:500	sc-26481	Santa cruz, USA
β-actin	mouse	42 kDa	1:2000	sc-47778	Santa cruz, USA

### Statistical analysis

All the experiments were repeated for three times and all data were showed as a mean ± standard deviation. Statistical evaluation was performed using *Mann–Whitney* U to differentiate the means of different groups. *P <* 0.05 was considered as statistically significant. SPSS 10.0 software was employed to analyze all data.

## Results

### The role of RhoC in EMT of ovarian carcinoma cells

**Figure 1:**
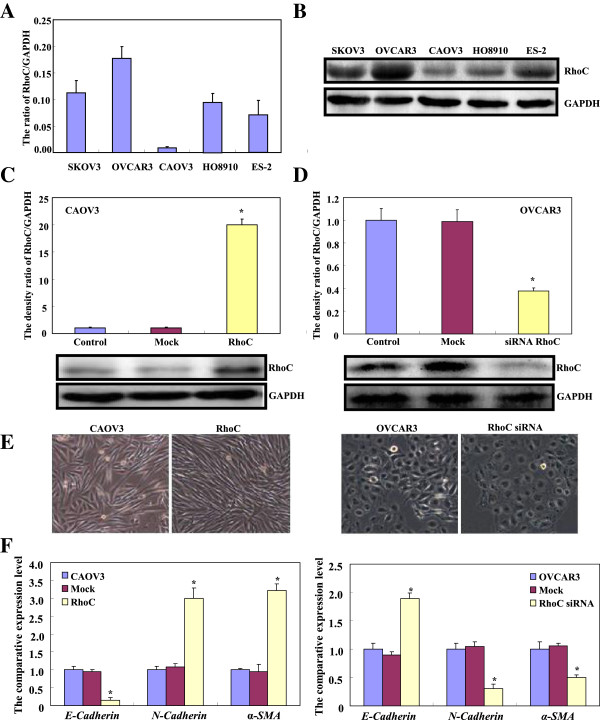
**The involvement of RhoC in EMT of ovarian carcinoma cells.** The mRNA and protein expression of RhoC was screened in ovarian carcinoma cells (SKOV3, OVCAR3, CAOV3, HO8910 and ES-2) by real-time PCR **(A)** and Western blot **(B)**. CAOV3 cells were transfected with RhoC-expressing plasmid and confirmed by real-time PCR and Western blot **(C)**. After transfection of RhoC siRNA, RhoC expression became weaker in OVCAR3 by real-time PCR and Western blot **(D)**. CAOV3 cells became spindle after ectopic RhoC expression, while RhoC knockdown caused OVCAR3 morphorlogically round **(E)**. There was a down-regulated expression of *E-cadherin* mRNA, and up-regulated expression of *N-cadherin* and *a-SMA* mRNA in CAOV3 transfectants by real-time PCR **(F)**. After the treatment of RhoC siRNA, there was an increased expression of *E-cadherin* mRNA in OVCAR3 cells by real-time PCR, while the converse was true for the expression of *N-cadherin* and *a-SMA* mRNA **(F)**. * compared with control and mock, p < 0.05.

As shown in Figure 1A and B, RhoC was strongly expressed in SKOV3, OVCAR3, HO8910, and ES-2, but weakly expressed in CAOV3 at both the mRNA and protein levels. Therefore, we selected CAOV3 for RhoC-expressing plasmid transfection and OVCAR3 for RhoC siRNA treatment. In comparison with the control and mock, RhoC overexpression was detected in CAOV3 cells after plasmid transfection at both the mRNA and protein levels (Figure 1C, p < 0.05). After siRNA treatment, RhoC expression became weaker in OVCAR3 transfectants than control and mock cells by real-time PCR and Western blot (Figure 1D, p < 0.05). Compared with the control and mock, siRNA transfectants had a round appearance under light microscopy, while plasmid transfectants displayed a spindle appearance (Figure 1E, p < 0.05). RhoC overexpression down-regulated *E-cadherin* mRNA expression and up-regulated *N-cadherin* and *a-SMA* mRNA expression in CAOV3 transfectants, compared with mock and control cells (Figure 1F). After RhoC siRNA treatment, *E-cadherin* mRNA expression was higher in OVCAR3 transfectants than control and mock cells by real-time PCR, while *N-cadherin* and *a-SMA* mRNA expression was lower (Figure 1F).

### RhoC-mediated effects of VEGF and TGF-β1 on EMT and related molecules in ovarian carcinoma cells

**Figure 2:**
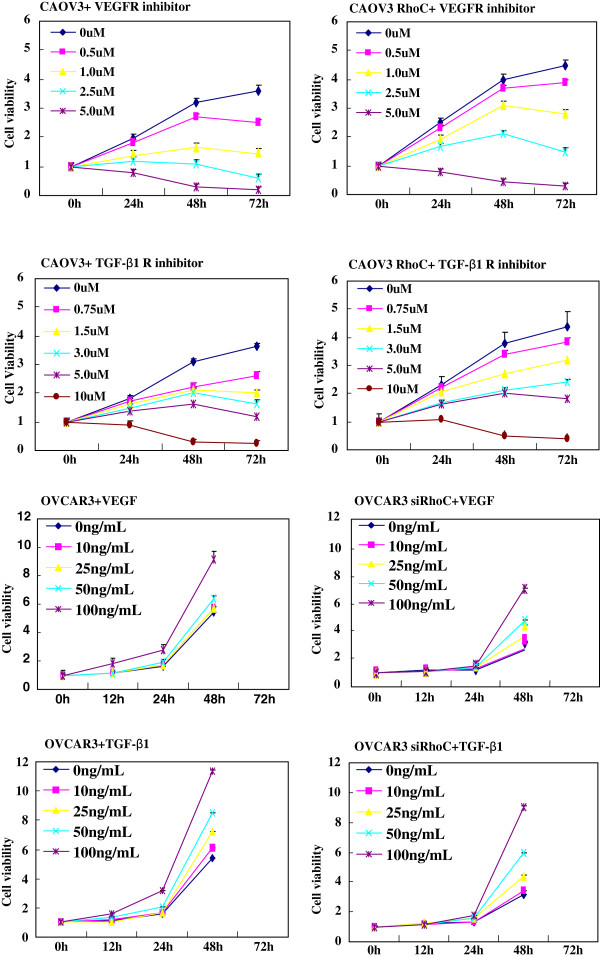
**The RhoC-mediated effects of TGF-β1 and VEGF on proliferation of ovarian carcinoma cells.** VEGFR or TGF-β1R inhibitor could suppress the proliferation of CAOV3 in both dose-dependent and time-dependent manners, but both factors promoted the proliferation of OVCAR3. VEGFR inhibitor (2.5 μM), TGF-β1R inhibitor (5.0 μM), VEGF (100 ng/mL) and TGFβ1 (100 ng/mL) were employed to treat these ovarian carcinoma cells in the following experiments of Figures 3, 4, 5, and 6.

**Figure 3:**
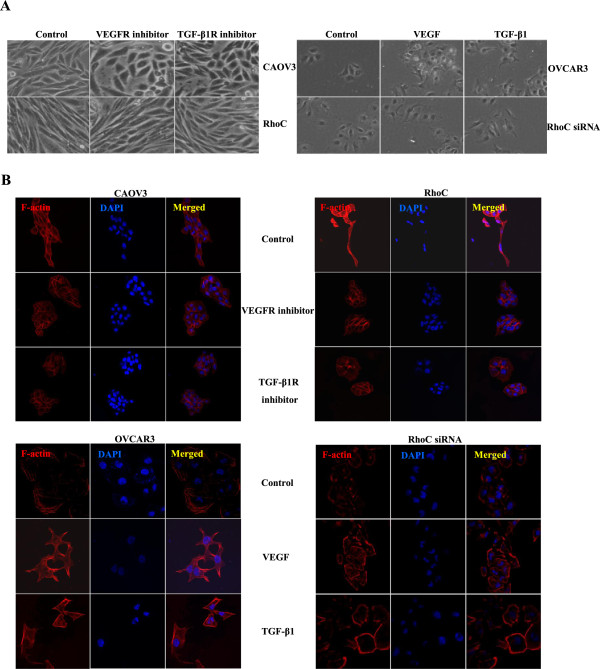
**The RhoC-mediated effects of TGF-β1 and VEGF on EMT and lamellipodia formation of ovarian carcinoma cells.** Morphologically, the treatment of VEGFR and TGF-β1R inhibitors might result in the increased ratio of round CAOV3 and transfectant cells, but the exposure to both growth factors could make more OVCAR3 and transfectant cells become spindle **(A)**. Both inhibitors could decrease the ability of CAOV3 cells or their transfectants to form lamellipodia by F-actin staining, while RhoC overexpresion could enhance the effect. Growth factors induced the OVCAR3 and RhoC siRNA transfectants to form lamellipodia, while RhoC knockdown caused the weaker ability of both cells **(B)**.

**Figure 4:**
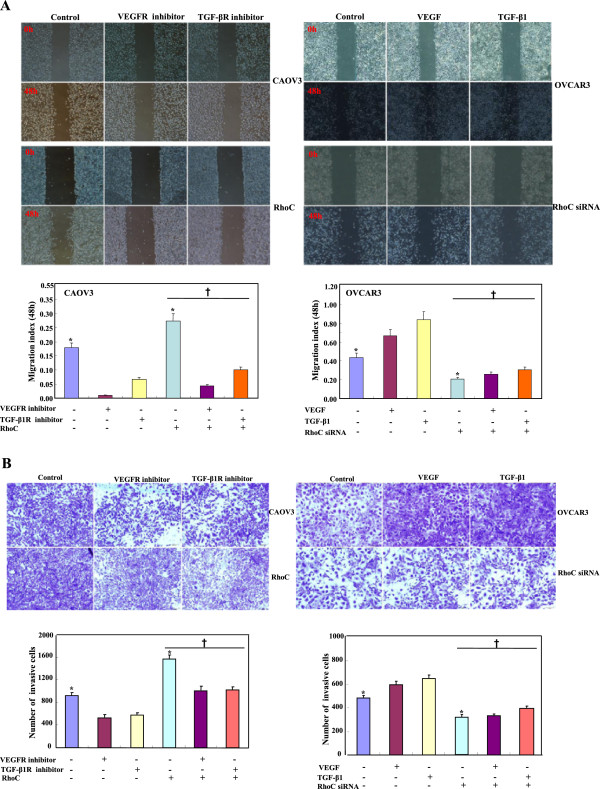
**The RhoC-mediated effects of TGF-β1 and VEGF on migration and invasion of ovarian carcinoma cells.** Inhibitors could decrease the ability of CAOV3 cells or their transfectants to migrate by wound healing assay **(A)** and invade by transwell **(B)**, while RhoC overexpresion could enhance the effects. Growth factors caused the OVCAR3 and RhoC siRNA transfectants to highly migrate **(A)** and invade **(B)**, while RhoC knockdown caused the weaker abilities of both cells **(A and B)**. * compared with treating groups, p < 0.05. † compared with corresponding either RhoC- overexpressing or -hypoexpressing group.

TGF-β1R or VEGFR inhibitors suppressed the proliferation of CAOV3 cells in both dose-dependent and time-dependent manners, but TGF-β1 or VEGF promoted proliferation of OVCAR3 cells and their transfectans (Figure 2). Exposure to both the receptor inhibitors increased the ratio of round CAOV3 cells and their transfectancts although both the growth factors caused elongation of OVCAR3 cells (Figure 3A). VEGFR or TGF-β1R inhibitors decreased the ability of CAOV3 cells and their RhoC transfectants to form lamellipodia (Figure 3B), migrate (Figure 4A, p < 0.05), and invade (Figure 4B, p < 0.05), while VEGF or TGF-β1 enhanced lamellipodia formation (Figure 3B, p < 0.05), migration (Figure 4A) and invasion (Figure 4B, p < 0.05) of OVCAR3 and their RhoC siRNA transfectants. Ectopic RhoC overexpression enhanced proliferation, migration, invasion and lamellipodia formation of CAOV3 cells regardless of the treatment of VEGFR or TGF-β1R inhibitor, whereas RhoC knockdown weakened above- mentioned biological events of OVCAR3 cells even with the exposure to VEGF or TGF-β1 (Figures 2, 3, and 4).

**Figure 5:**
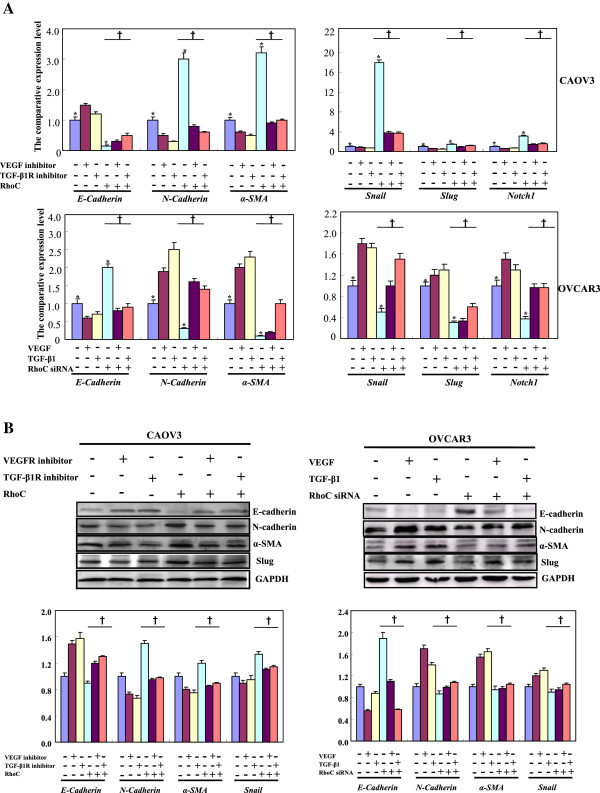
**The RhoC-mediated roles of VEGF and TGF-β1 in the expression of EMT-related molecules.** In CAOV3 cells, VEGFR and TGF-β1R inhibitors could up-regulate *E-cadherin* mRNA expression and down-regulated *N-cadherin*, *α-SMA*, *snail* and *Notch1* mRNA expression, but their growth factors had the opposite effects in OVCAR3 cells by real- time PCR **(A)**. According to Western blot and densitometric analysis, both inhibitors increased the E-cadherin expression, but decreased N-cadherin, α-SMA and Slug expression **(B)**. Growth factors suppressed the E-cadherin expression, while enhanced the expression of N-cadherin, α-SMA and Slug **(B)**. RhoC overexpression decreased the E-cadherin expression and increased the expression of N-cadherin, α-SMA, Slug and Notch1 in CAOV3 cells, while RhoC siRNA had the opposite effects in OVCAR3 cells **(A and B)**. * compared with treating groups, p < 0.05; † compared with corresponding either RhoC- overexpressing or -hypoexpressing group.

**Figure 6:**
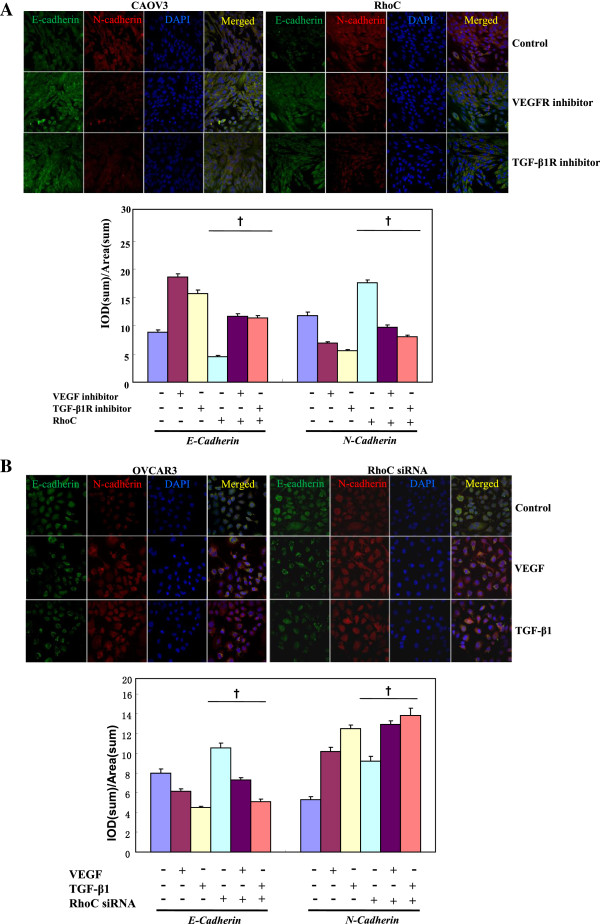
**The RhoC-mediated roles of VEGF and TGF-β1 in the expression of E-cadherin and N-cadherin by immunofluorescence.** In CAOV3 cells, VEGFR and TGF-β1R inhibitors could up-regulate the E-cadherin expression and down-regulated N-cadherin expression **(A)**, but their growth factors had the opposite effects in OVCAR3 cells **(B)**. RhoC overexpression decreased the E-cadherin expression and increased the expression of N-cadherin in CAOV3 cells, while RhoC siRNA had the opposite effects in OVCAR3 cells **(A and B)**. * compared with treating groups, p < 0.05; † compared with corresponding either RhoC- overexpressing or -hypoexpressing group.

In CAOV3 and its RhoC transfectant, VEGFR and TGF-β1R inhibitors up-regulated *E-cadherin* mRNA expression and down-regulated *N-cadherin*, *α-SMA*, *snail* and *Notch1* mRNA expression, but corresponding growth factors had the opposite effects in OVCAR3 and RhoC- knockdown transfectants based on real-time PCR (Figure 5A, p < 0.05). E-cadherin expression was increased and N-cadherin, α-SMA and Slug expression were decreased in CAOV3 and its transfectants treated by receptor inhibitors. Growth factors inhibited E-cadherin expression, while promoting N-cadherin, α-SMA and Slug expression (Figure 5B, p < 0.05). Immunofluorescence results for E- and N-cadherin were similar to those shown by Western blot (Figure 6, p < 0.05). RhoC overexpression decreased the expression of the epithelial markers (E-cadherin) and increased mesenchymal markers (N-cadherin, α-SMA, Slug and Notch1) in CAOV3 cells even exposed to VEGFR or TGF-β1R inhibitor. In contrast, RhoC siRNA had the opposite effects in OVCAR3 cells, treated with or without VEGF or TGF-β1 (Figures 5 and 6, p < 0.05).

## Discussion and conclusions

As reviewed, a possible role for RhoC was clarified in the EMT-related invasion and in metastasis because *in vivo* and *vitro* RhoC overexpression is associated with tumor cell invasion and metastasis [7]. In colon carcinoma, RhoC protein expression and subsequent activation were detected coincident with the loss of E-cadherin and acquisition of mesenchymal characteristics. A marked increase in RhoC expression was associated with the EMT of colon carcinoma cells and RhoC promoted post-EMT cell migration [14]. Here, we found the promoting effects of RhoC in EMT of ovarian carcinoma cells, evidenced by the alteration in morphological appearance and EMT markers (E-cadherin, N-cadherin and α-SMA) in either RhoC-overexpressing or –hypoexpressing cells. In line with previous reports [16,17], forced RhoC overexpresion resulted in the faster migration, higher invasion and more lamellipodia formation for ovarian carcinoma cells, while RhoC knockdown did the opposite. In particular, our previous study demonstrated that the treatment with either RhoC siRNA or Rho inhibitor, Lovastatin reduced the mobility of ovarian carcinoma cell, OVCAR3, possibly through the down-regulation of MMP-9 and VEGF [9,10]. These data suggested that RhoC might be a signaling protein in the EMT pathway of ovarian carcinoma cells.

Various reports showed that TGF-β1 and VEGF might initiate the EMT of carcinoma cells [18-20]. In the present study, it was found that both TGF-β1R and VEGFR inhibitors decreased the aggressive phenotypes (e.g. proliferation, migration, invasion and lamellipodia formation) in CAOV3 and its RhoC transfectants. In contrast, both TGF-β1 and VEGF had the converse biological effects in OVCAR3 and RhoC-knockdown transfectants. Interestingly, RhoC siRNA might inhibit migration, invasion and lamellipodia formation of OVCAR3 treated with or without TGF-β1 or VEGF, while RhoC overexpression might promote these events of CAOV3 cells even with the exposure to TGF-β1R or VEGFR inhibitor. Mukai et al. [21] demonstrated that RhoC overexpression plays a critical role in the migration of hepatoma cells in rat ascites after the treatment of TGF-β1. Wang et al. [22] showed that RhoC is the downstream regulator of VEGF in endothelial cells and is essential for angiogenesis induced by VEGF. These indicated that VEGF and TGF-β1 might promote the migration, invasion and EMT of ovarian carcinoma cells, which is possibly regulated by RhoC.

To explore the molecular mechanisms about the role of VEGF and TGF-β1 in EMT of ovarian carcinoma cells, we examined the EMT-related molecules in combination with quantitative PCR, Western blot and immunofluorescence. Consequently, it was found that both recombinant VEGF and TGF-β1 could down-regulate *E-cadherin* expression, but up-regulate *N-cadherin* and *α-SMA* expression with the opposite role of both their receptor inhibitors, supporting the regulatory effects of VEGF and TGF-β on EMT of ovarian carcinoma cells. During EMT, the exposure to TGF-β1 might up-regulate *Snail* and *Slug* expression and increase cell invasion [23]. The canonical TGF ß-Smad signaling might also regulate *Snail* and *Slug* expression [24]. Here, the exposure to VEGF or TGF-β1 increased snail expression at both mRNA and protein levels, indicating RhoC also promote the event of EMT as a signal molecule. According to the literature, the activation of Notch-1 signaling contributes to the acquisition of EMT phenotype of pancreatic carcinoma cells [25]. Another study has provided evidences for the opinion that RhoC is an effector of Notch1 in cervical carcinoma cells [12]. Here, it was worth noting that VEGF and TGF-β1 also enhanced Notch1 expression *via* RhoC protein, which will form a positive feedback loop for the initiation of EMT. After RhoC-expressing plasmid transfection, there appeared the down-regulated expression of the epithelial markers and the up-regulated expression of mesenchymal markers in CAOV3 cells regardless of the exposure to VEGFR or TGF-β1R inhibitor. In contrast, RhoC siRNA caused the opposite effects in OVCAR3 cells, even treated with both VEGF and TGF-β1. Taken together, VEGF and TGF-β1 were suggested to play an important role in EMT of ovarian carcinoma cells possibly *via* RhoC and final effectors, including snail and slug.

In summary, our study indicated that aberrant RhoC expression might be involved in EMT of ovarian cancer cells, initiated by TGF-β1 and VEGF. The above-mentioned three molecules should be considered as good targets to reverse EMT of ovarian carcinoma cell, which is useful and helpful for the treatment of the metastasis and recurrence of ovarian carcinoma.

## Competing interests

The authors have declared that no competing interests exist.

## Authors’ contributions

HCZ designed the study and wrote the manuscript. WFG, YZ, HL, XFY, YLX, SZ, JML, ZTZ and HZS finished the experiments of cell culture, molecular and morphological examination, and animal model. FX, YPL and YT helped us with statistical analysis, English checking and manuscript correction. All authors read and approved the final manuscript.

## Pre-publication history

The pre-publication history for this paper can be accessed here:

http://www.biomedcentral.com/1471-2407/14/477/prepub
